# VP4/VP56/VP35 Virus-like Particles Effectively Protect Grass Carp (*Ctenopharyngodon idella*) against GCRV-II Infection

**DOI:** 10.3390/vaccines11081373

**Published:** 2023-08-16

**Authors:** Qingqing Tian, Xingchen Huo, Qian Liu, Chunrong Yang, Yongan Zhang, Jianguo Su

**Affiliations:** 1 Hubei Hongshan Laboratory, College of Fisheries, Huazhong Agricultural University, Wuhan 430070, China; tqq13271507495@webmail.hzau.edu.cn (Q.T.); huoxingchen2020@webmail.hzau.edu.cn (X.H.); liuqian2021.hzau.edu@webmail.hzau.edu.cn (Q.L.); yonganzhang@mail.hzau.edu.cn (Y.Z.); 2 Laboratory for Marine Biology and Biotechnology, Pilot National Laboratory for Marine Science and Technology, Qingdao 266237, China; 3 College of Veterinary Medicine, Huazhong Agricultural University, Wuhan 430000, China; chryang@mail.hzau.edu.cn

**Keywords:** grass carp (*Ctenopharyngodon idella*), virus-like particles, grass carp reovirus, vaccine, *Pichia pastoris* expression, Astragalus polysaccharide

## Abstract

**Highlights:**

*1.*  *Three outer capsid proteins of GCRV-II self-assemble into VLPs in vitro.*
*2.*  *VLPs vaccine have low toxicity and uniform particle size.*
*3.*  *VLPs + astragalus polysaccharide improve protection and reduces tissue viral load.*
*4.*  *VLPs vaccine induce immune response and regulate antioxidant immunity.*

**Abstract:**

Grass carp reovirus (GCRV) seriously threatens the grass carp (*Ctenopharyngodon idella*) industry. Prophylactic GCRV vaccines prepared by virus-like particle (VLP) assembly biotechnology can improve effectiveness and safety. The highly immunogenic candidate antigens of GCRV vaccines that have been generally considered are the outer capsid proteins VP4, VP56, and VP35. In this study, VP4, VP56, and VP35 were expressed in an *Escherichia coli* expression system and a *Pichia pastoris* expression system. The successful assembly of uniform, stable, and non-toxic VP4/VP56/VP35 VLPs was confirmed through various assays. After vaccination and GCRV infection, the survival rate in the VLPs + adjuvant Astragalus polysaccharide (APS) group was the highest (62%), 40% higher than that in control group (22%). Through the antibody levels, tissue viral load, and antioxidant immunity assays, the *P. pastoris* VLP vaccine effectively improved IgM levels, alleviated tissue virus load, and regulated antioxidant immune-related indicators. The treatment with *P. pastoris* VLPs enhanced the mRNA expression of important immune-related genes in the head kidney, as measured by qRT-PCR assay. Upon hematoxylin-eosin staining examination, relatively reduced tissue pathological damage was observed in the VLPs + APS group. The novel vaccine using *P. pastoris* VLPs as an effective green biological agent provides a prospective strategy for the control of fish viral diseases.

## 1. Introduction

Grass carp (*Ctenopharyngodon idella*), a pivotal piscine species in the realm of aquaculture, carries considerable importance within China’s freshwater fish culture. It is distinguished by its considerable economic viability, exceptional growth performance, delectable umami meat quality, and heightened nutritional value [1]. Nonetheless as a consequence of intensive farming advancements, the emergence of disease-related challenges has become an arduous endeavor in the cultivation of grass carp [2,3]. Grass carp reovirus (GCRV) serves as the etiological agent responsible for inducing grass carp hemorrhagic disease (GCHD), which presents a grave peril to the grass carp aquaculture industry [4,5]. Confronted with the formidable pathogenicity and wide-ranging infectivity exhibited by GCRV, there exists an urgent necessity to develop a biological agent capable of combating GCRV infection.

Vaccination represents one of the most efficacious approaches to disease prevention [5,6]. In recent years, virus-like particle (VLPs) vaccines have garnered considerable acclaim owing to their intricately structured, hollow particles that are composed of viral structural proteins that possess the ability to self-assemble [7]. In comparison with conventional attenuated and inactivated vaccines, VLPs offer notable advantages as immunogens. These VLPs have exhibited physicochemical properties akin to natural viruses, yet lack genetic material, thereby rendering them highly safe. Moreover, antigen-presenting cells can effectively process and modify VLPs, thereby eliciting both humoral and cellular immune responses and affording heightened immunogenicity [8,9,10]. VLPs formed by immunogenic antigens of exceptional potency are typically regarded as promising candidate vaccines, gaining increasing prominence in the realm of viral diseases prevention [11]. Consequently, recombinant VLPs have emerged as an enticing prospect for the design of GCRV vaccines.

Various cell culture systems have been employed for the production of recombinant VLPs, yielding successful outcomes in vaccine manufacturing [12]. Yeast-based expression systems, in particular, offer a multitude of advantages, including cost-effectiveness, the secretion of expressed proteins, eukaryotic modifications, rapid growth, and being generally recognized as safe (GRAS) [13,14]. Notably, several VLP vaccines have been developed utilizing yeast systems, such as the recombinant-yeast-derived human hepatitis B vaccine [15]. Preclinical and early clinical studies have demonstrated the immunogenicity of purified VLPs produced through *Saccharomyces cerevisiae* expression, utilizing HPV16L1-derived VLPs [16]. Another widely employed expression system in the field of biomedicine is *Escherichia coli*, known for its simplicity, mature technology, cost-effectiveness, high expression levels, and safety [17]. Recombinant rotavirus VLPs have also been expressed in *E. coli* and assembled in vitro, with subsequent investigations into their characteristics and immunogenicity [8]. Previous studies have provided evidence of virion-like assembly, suggesting that VLPs can be assembled through similar methods, thereby offering potential as vaccines or therapeutic agents for combating viral infections [18].

The robust immunogenicity of the three key outer capsid proteins, (VP4, VP56, and VP35) found in GCRV, positions them as promising antigenic candidates. Previous research has revealed that the S6, S7, and S11 genes of GCRV-II encode VP4, VP56, and VP35, respectively [19]. The S6 gene, spanning a length of 2028 bp, features a lengthy single ORF on its plus strand, encoding the primary outer capsid protein VP4. VP4 assumes a critical role in both viral assembly and cellular invasion. Due to its favorable antigenicity, the S6 gene is commonly employed in subunit vaccine studies [20,21,22]. Encoded by the S7 segment, the VP56 protein exhibits a fibrillar structure specific to GCRV-II and plays a significant role in adhering to the host cell surface during GCRV-II infection [4,21]. The VP35 protein, originating from the S11 gene fragment was also identified [23]. VP4, VP56, and VP35 hold potential as candidate vaccine antigens as they are capable of eliciting immune responses within the host.

Co-administration of fusion proteins and adjuvants represents a potential strategy to augment the immunological potency of vaccines. Presently, there is growing interest in utilizing Chinese herbal medicines as vaccine adjuvants [24]. Astragalus polysaccharide (APS), derived from the Chinese herb *Astragalus membranaceus*, has been the subject of previous investigations due to its diverse biological and pharmacological activities, encompassing antioxidative, antitumor, immunomodulatory, and antiviral effects. Research reports have indicated that APS possesses the capability to modulate immune activity and other immune functions in fish, thereby functioning as an adjuvant to enhance vaccine expression and bolster immune efficacy [24,25,26,27].

In addition to the inherent complexities associated with VLP production, assessing the effectiveness of candidate vaccines can present challenges. Previous studies have indicated that the non-enteric route of administration, specifically injection, has been favored for vaccines [28].This mode of administration offers prompt efficacy and elicits a robust immune response that accurately reflects the antigen-specific immune response [1]. Considering VLPs as a novel type of antiviral vaccine, their immune effects in fish can be more comprehensively and thoroughly investigated through injection. Therefore, the injection may serve as a more dependable and informative approach for evaluating the efficacy of VLP-based vaccines in fish.

This research explicates the generation and assembly of homogeneous VLPs employing purified capsid proteins through the expression systems of *E. coli* and *P. pastoris*. A VLP production methodology was devised, encompassing the analysis of morphological and physicochemical properties. The optimal immunization concentration was determined, and adjuvants were incorporated into the VLP vaccines formulation. Subsequently, the vaccine was administered intramuscularly to immune grass carp to evaluate its protective efficacy against GCHD. This innovative approach, involving the selection and optimization of VLP-based vaccines in conjunction with the incorporation of adjuvants, represents a significant advancement in the prevention and control of GCHD. The strategy exhibited promise in effectively averting and managing viral diseases in aquaculture, constituting a noteworthy contribution to the domain of aquatic animal health management.

## 2. Materials and Methods

### 2.1. Experimental Animals, Bacteria, Virus, and Gene Cloning of Full-LengthVP4/VP56/VP35 

Healthy grass carp (weight = 20 ± 5 g) were derived from a fish farm located in Wuhan, Hubei Province, China. The healthy fish were randomly assigned to six experimental groups (50/group), and each tank was filled with 200 L of water and maintained at 28–30 °C. The animals were acclimated for a minimum of 2 weeks before the experiments, and the experimental fish were fed twice daily. All animal experiments were approved by the Ethical Committee on Animal Research at Huazhong Agricultural University (HZAUFI-2021-0026). All efforts were aimed at minimizing the suffering of animals.

The BL21(DE3) (Invitrogen, Carlsbad, CA, USA) used to express the recombinant protein was stored in glycerol at −80 °C, and cultivated in Luria-Bertani (LB) liquid medium at 37 °C. Similarly, the *P. pastoris* GS115 strain stored in our laboratory was kept at −80 °C. The GS115 strain was cultivated in yeast extract peptone dextrose (YPD) liquid medium at 28 °C.

The GCRV-II strain GCRV 097 was preserved in our laboratory. GCRV 097 was propagated in *C. idella* kidney (CIK) cells at 28 °C with 5% CO_2_, in DMEM (Gibco, Beijing, China) medium supplemented with 10% FBS (Gibco, Beijing, China), 100 U/mL penicillin (Sigma, Shanghai, China), and 100 U/mL streptomycin (Sigma, Shanghai, China).

The total RNA of GCRV-II was extracted and the concentration and purity determined using a NanoDrop 2000 spectrophotometer (Thermo Scientific, Waltham, MA, USA). The quality of the RNA was also evaluated through 1.5% agarose gel electrophoresis. The mRNA was further reverse-transcribed into cDNAs with MMLV reverse transcriptase, an RNase inhibitor (Thermo Fisher Scientific, Waltham, MA, USA), and a hexamer random primer. PCR amplification (with primers listed in Table 1) was conducted to obtain the full-length encoding of VP4, VP56, and VP35. The amplification was performed on a ProFlex™ PCR system with the following conditions: 95 °C for 5 min, followed by 35 cycles at 95 °C for 30 s, 56 °C for 30 s, 72 °C for 1 min, and the final extension at 72 °C for 10 min. Lastly, the PCR products underwent purification using a DNA gel purification kit (Axygen, Hangzhou, China).

### 2.2. Recombinant Expression and Purification of the VP4/VP56/VP35 Proteins in Prokaryotic and Yeast Systems

To express and purify the recombinant proteins, purified PCR products were first digested with the restriction enzymes *BamH I* and *Xho I* and then ligated into the respective expression vectors. For the pGEX-4T-1 expression vector, recombinant proteins were expressed in BL21 (DE3) cells and purified using glutathione s-transferase-nitrilotriacetic acid (GST-NTA) beads. Meanwhile, the purified PCR products were digested with the restriction enzymes *BamH I* and *Xho I* and then ligated into the pET-28a (+) expression vector. The recombinant proteins were expressed in BL21 (DE3) cells and purified using Ni-nitrilotriacetic acid (Ni-NTA) beads. After crushing the bacterial sample and centrifuging, the supernatant was collected and subjected to affinity chromatography using GST-NTA and Ni-NTA, respectively. The mixture was washed with a washing solution (His: 10 mM imidazole, 20 mM Tris-HCl, 500 mM NaCl, 10% glycerol, pH 7.5; GST: 20 mM Tris-HCl, 400 mM NaCl, 10% glycerol, pH 7.5) and then eluted with an elution buffer (300 mM imidazole/10 mM GSH, 20 mM Tris-HCl, 200 mM NaCl, pH 7.5). Imidazole was removed from the collected protein solution through dialysis and concentrated through ultrafiltration to obtain the final protein.

To express VP4/VP56/VP35 using a yeast expression system, the DNA sequences of VP4/VP56/VP35 were modified by linking a 6 × His-tag sequence at their ends. Subsequently, the modified sequences were inserted into the pPIC9K (p9K) plasmid to generate pPIC9K-VP4 (His-VP4), pPIC9K-VP56 (His-VP56), and pPIC9K-VP35 (His-VP35), respectively. The empty vector was utilized as a negative control. The linearization of His-VP4, His-VP56, and His-VP35 was carried out using *Sal I* and the products were transformed into *P. pastoris* GS115 via electroporation (7000 V/cm, 25 μF, ×400; Life Technologies Cell-Porator in Carlsbad, CA, USA). Subsequently, 1 mL of sorbitol solution was added to the electroporated cells, which were incubated for 1 h at 28 °C and 200 rpm to allow the recovery of yeast cells. The above-mentioned solution was then spread on MD plates (pH 6.0, containing 2% sucrose, 1.5% agar, 1.34% YNB, and 0.00004% biotin) and YPD + G418 plates (containing 1% yeast extract, 2% peptone, 2% dextrose, 2% agar, and 0.2–0.8 mg/mL G418). The plates were incubated at 30 °C for approximately 48–72 h to select well-growing transformant colonies. The selected colonies were inoculated into 100 mL of buffered glycerol-complex medium (BMGY; containing 1% yeast extract, 2% peptone, 1.34% YNB, 0.00004% biotin, 1% glycerol, 100 mM potassium phosphate buffer, pH 6.0) and cultured at 28 °C and 200 rpm until reaching the logarithmic growth phase (OD600 approximately 1.0). The cells were then collected by centrifugation, the culture medium was removed, and the cells were resuspended in the same volume of buffered minimal methanol YP medium (BMMY; the same as BMGY but replacing glycerol with 0.5% methanol). Methanol was used as the inducer at a concentration of 0.5–1.0%. The concentration and duration of the inducer should be optimized according to experimental requirements and the induction characteristics of the target protein. The culture was further incubated at 28 °C and 200 rpm for 24–72 h under the presence of the inducer. The p9K followed the same experimental procedure. Furthermore, as pPIC9K was used for protein secretion, the proteins, His-VP4, His-VP56, and His-VP35, in the supernatant were isolated and purified using the His-tag Protein Purification Kit (P2226; Beyotime, Haimen, China). We took 15 mL supernatant and chromatographed it directly using a Ni-NTA affinity chromatography column. After washing with a cleaning solution (20 mM imidazole, 20 mM Tris-HCl, 500 mM NaCl, 10% glycerol, pH 7.5), elution was performed with an elution buffer (300 mmol/L imidazole, 20 mM Tris-HCl, 300 mM NaCl, pH 7.5). The collected protein solution was dialyzed to remove imidazole, and the final protein was obtained through ultrafiltration concentration. Finally, the protein concentrations were determined using the Bradford method.

### 2.3. SDS-PAGE Analysis and Western Blot (WB)

The recombinant protein VP4/VP56/VP35 was analyzed on 10% SDS-PAGE and by WB. The protein samples were separated by 10% SDS-PAGE and the gels were stained using Coomassie Brilliant Blue G-250 (C.I.42655, Sigma, Shanghai, China). For the WB, the gels were transferred onto a nitrocellulose (NC) filter membrane (Millipore, Burlington, MA, USA). The membrane was then blocked with 5% skim milk in phosphate-buffered solution-Tween-20 (PBST, Boster, Wuhan, China) for 2 h and washed with PBST. Afterward, the mouse anti-His-tag/anti-GST-tag monoclonal antibody (1: 3000, ABclonal, Wuhan, China, AE003/AE001) was added and the incubation continued for 2 h. The membrane was then rewashed with PBST and incubated with a 1: 2000 dilution of horseradish peroxidase (HRP)-conjugated goat anti-mouse IgG antibody (ABclonal, Wuhan, China, AS064) for 1 h. Following another wash with TBST, the NC filter membrane was stained with Clarity TM Western ECL Substrate (Bio-Rad, Hercules, CA, USA) and y imaged using the Amersham Imager 600 (Little Chalfont). Finally, the purified proteins and a control sample (10 μL culture supernatant of GS115 transfected with p9K) were also detected using the same method.

### 2.4. Assembly of GCRV Virus-like Particles (VLPs)

GCRV-VLPs were assembled by allowing the purified VP4/VP56/VP35 protein to incubate in 1 L assembly solution with different NaCl concentrations, pH and 20 mM phosphate/40% saturated (NH_4_)_2_SO_4_ buffer at 4 °C for at least 36 h. The assembly fluid was changed every six hours.

### 2.5. Pull-Down, Transmission Electron Microscopy (TEM), and Dynamic Light Scattering (DLS) Analysis

The VLP solution was transferred to a fresh tube, and incubated with GST-bind resin/anti-VP56 rabbit for 2 h at 4 °C. The beads were collected by centrifugation at 3000× *g* for 5 min and thoroughly washed three times with TBST. Subsequently, the beads were suspended in 20 μL 2 × SDS loading buffer, denatured at 95 °C for 10 min, and then analyzed by SDS-PAGE and subsequent Western blotting.

The VLP samples were prepared for analysis by applying them to a carbon-coated copper grid, which was then negatively stained with 2% phosphotungstic acid (pH 5.0) for 30 s. Transmission electron microscopy (TEM, HT-7700, Hitachi, Japan) was used to view the samples.

To determine the VLP size in solution, a Malvern Nano-ZS 90 laser particle size analyzer (Malvern Instruments, Royston, UK) was used. The VLP solution (100 g/mL) was centrifuged at 17,000× *g* for 10 min before analysis. A total of 2 mL of the sample was then applied to the cuvette, and detection began once the temperature equilibrated to 25 °C.

### 2.6. Hemolysis and Cytotoxicity Tests

To test the potential toxicity of the VLPs, a hemolysis assay was conducted on grass carp red blood cells. Fresh peripheral blood (3 mL) was collected using sodium heparin tubes, and red blood cells were isolated through centrifugation (4 °C, 500 g, 10 min), flushed with PBS (pH = 7.4), and resuspended. In a 96-well plate, 100 μL of the VLPs and red blood cells were added, and the plate was then incubated at 37 °C for 2 h, followed by centrifugation (4 °C, 500 g, 10 min). The absorbance at 450 nm was measured for each well using a microplate reader. The MTT assay was used to evaluate the cytotoxicity of VLPs towards CIK and FHM cells. The cells were inoculated with VLPs in 96-well plates (1.0 × 10^4^ per well), treated with varying concentrations of the VLPs, and co-cultured at 37 °C for 1 h. The cell culture medium was replaced with 10% MTT solution, and after incubation at 37 °C and 5% CO_2_ for 4 h, the absorbance was measured at 595 nm using a microplate reader.

### 2.7. Injection Immunization and Viral Challenge

Grass carp were divided into six groups. There were 100 grass carp in each group, 50 of them were used to measure mortality, and the other 50 were used for sampling. Grass carp were injected with 40 µg VLPs (Vaccine group), 50 µg APS + 40 µg VLPs (APS–Vaccine group), 50 µg APS (APS group), 100 µL VP4/VP56/VP35 protein mixture solution (Mix group), 50 µg APS + 100 µL VP4/VP56/VP35 protein mixture solution (APS–Mix group), or 100 µL PBS (Control group, pH = 7.4). Fish were sacrificed for harvesting serum, head kidney, spleen tissues, and intestines on D1, D3, D7, D10, D14, D16, D21, and D28 post-immunization (*n* = 4). Tissues were collected to analyze the mRNA expression of IL-1β, IFN1, IFN-γ2, MHC-IIα, TNFα, CD4, and IgM.

To test the efficacy of the encapsulated VLP vaccine for grass carp against GCRV-II infection, a challenge test was conducted (all fish were injected). Intraperitoneal injection was found to be the optimal method for delivering the virus to the experimental fish, as determined through preliminary experiments. On day 15 post-vaccination, the fish were intraperitoneally challenged with GCRV-097 (1.0 × 10^6^ TCID_50_ mL^−1^). The water temperature of each tank was maintained at 28 °C using heating rods. From day 1 to day 14 post-challenge, mortality rates were monitored and recorded.

### 2.8. Enzyme Activity Assay in Serum, Quantitative RT-PCR Analysis of Immune-Related Gene Expression, Viral Load Determination and Statistical Analysis

The fish were anesthetized with MS-222 and blood samples were collected from their caudal vein using a 1 mL syringe. These blood samples were placed at 25 °C for 2 h and then subjected to centrifugation (4500 rpm/min, 10 min) (Multifuge X1R, Thermo fisher Scientific, Waltham, MA, USA) at 4 °C. The serum samples were collected after centrifugation and stored at −80 °C in our laboratory until further analysis. Various serum biochemical indices, including complement 3 (C3), total superoxide dismutase (T-SOD), malondialdehyde (MDA), catalase (CAT), total antioxidant capacity (T-AOC), and reactive oxygen species (ROS) were evaluated using commercial kits (Nanjing Jiancheng Bioengineering Institute, Nanjing, China). Detection was performed on sera obtained from four grass carp from each group.

To obtain total RNA, the TRIzol reagent (Aidlab, Beijing, China) was employed on head kidney samples following the manufacturer’s guidelines. RNA quantity was assessed using absorbance at 260 and 280 nm, and electrophoresis in 1.5% agarose gel was utilized to assess RNA integrity. Next, the mRNAs were individually reverse-transcribed into cDNA using HiScript^®^ II Q RT SuperMix for qPCR (+ gDNA wiper) (R223-01, Vazyme Biotech Co., LTD, Nanjing, China). The immune gene (IFN1, IFN-γ2, MHC-IIα, CD4, TNFα, and IL-1β) primers for performing qRT-PCR analyses along with their GenBank accession numbers are provided in Table 1;the reference control genes used were 18S rRNA and β-actin. The CT method (2^−ΔΔCT^) was employed to calculate the relative mRNA expression levels.

After 14 days of challenge, the viral load in grass carp tissues was analyzed using qRT-PCR with VP56-F and VP56-R primers (Table 1). The method of cDNA extraction from the intestine, head kidney, and liver samples of four fish from each experimental group remained the same as described previously. To conduct qRT-PCR, the following steps were performed: an initial step at 95 °C for 3 min, followed by 40 cycles of 95 °C for 15 s, 60 °C for 15 s, and 72 °C for 20 s.

The results were analyzed using GraphPad Prism 8.0 software and presented as mean ± standard deviation (SD). The Kruskal-Wallis test was used for statistical analysis of experimental data from each group, followed by Dunn’s multiple comparison test (with Bonferroni adjustment) to determine significance (*p* < 0.05). Different superscript letters (a, b, and c) are used to indicate significant variations within each group. The Mantel-Cox test was used to analyze protection rates. All results are presented as mean ± SD.

### 2.9. Hematoxylin and Eosin Staining

On D18, the gill, spleen, trunk kidney, and grain were collected from the fish and immediately fixed in 10% neutral buffered formalin for 24 h. The tissue samples were then embedded in paraffin, and four-micrometer-section samples were mounted on aminopropyl triethoxysilane-coated slides. Following deparaffinization in xylene, the sections were rehydrated and stained with hematoxylin and eosin (HE). Afterwards, they were mounted with neutral gum, and images were captured using the SQS-40P system (Shenzhen, China).

## 3. Results

### 3.1. Protein Expression, Purification, and Particle self-Assembly Analysis of VP4/VP56/VP35

**Figure 1:**
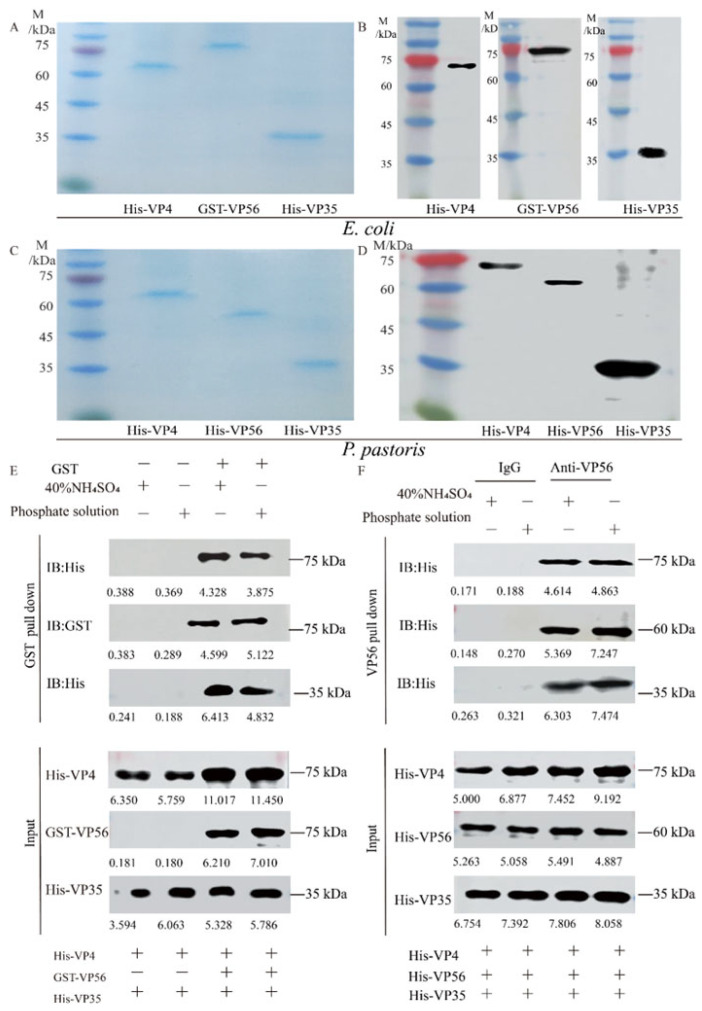
The expression, purification, and self-assembly verifying VP4, VP56, and VP35 proteins. (**A**) SDS-PAGE analysis of the VP4, VP56, and VP35 proteins after expression and purification by *E. coli*. (**B**) Western blot analysis of VP4, VP56, and VP35 proteins. The His-VP4 (67 kDa), GST-VP56 (81 kDa), and His-VP35 (36 kDa) proteins detected using GST mAbs or His mAbs as the primary antibody and horseradish-peroxidase-labeled goat anti-mouse IgG HRP as the secondary antibody. (**C**) SDS-PAGE analysis of the VP4, VP56, and VP35 proteins after expression and purification by *P. pastoris*. (**D**) Western blot analyses of VP4, VP56, and VP35 proteins. The His-VP4 (69 kDa), His-VP56 (59 kDa), and His-VP35 (36 kDa) proteins were detected using His mAbs as the primary antibody and horseradish peroxidase-labeled goat anti-mouse IgG HRP as the secondary antibody. (**E**,**F**) The VP4, VP56, and VP35 binding verified by pull-down analysis. *E. coli* protein samples and *P. pastoris* protein samples in 20 mM phosphate or 40% saturated (NH_4_)_2_SO_4_ buffer were used for incubation at 4 °C for 36 h.

Both prokaryotic VP4/VP56/VP35 proteins and eukaryotic VP4/VP56/VP35 proteins were successfully expressed (Figure S1A–D) and purified to a state of homogeneity (Figure 1A,C) respectively. WB analysis revealed the presence of specific bands at 67 kDa, 81 kDa, and 36 kDa for His-VP4, GST-VP56 and His-VP35, respectively (Figure 1B), and69 kDa, 59 kDa, and 36 kDa for His-VP4, His-VP56, and His-VP35, respectively (Figure 1D). Through testing various buffer conditions, the optimal conditions for their assembly were determined. The binding behavior of the proteins was confirmed through pull-down experiments (Figure 1E,F). Upon evaluating the screening results, buffers representing A (20 mM phosphate buffer, pH 6.0, with 100 mM NaCl) and B (40% saturated (NH_4_)_2_SO_4_ buffer, pH 7.0, with 100 mM NaCl) conditions were detected for the assembly of virus-like particles (Figure 1E,F) Specifically, the pull-down experiments demonstrated the binding of His-VP4, GST-VP56, and His-VP35 proteins to GST tags, which was further confirmed through Western blotting experiments (Figure 1E); Furthermore, the pull-down experiments confirmed that the His-VP4, His-VP56, and His-VP35 proteins could aggregate and bind with VP56-specific polyclonal antibodies, resulting in polymer formation, as visualized through SDS-PAGE (Figure 1F). These findings provided evidence of the purification of proteins with high purity and demonstrated the self-assembly properties and multimerization of viral capsid proteins in vitro under specific conditions.

### 3.2. VLPs Are Morphologically Stable, Uniform in Size, and Low in Toxicity

**Figure 2:**
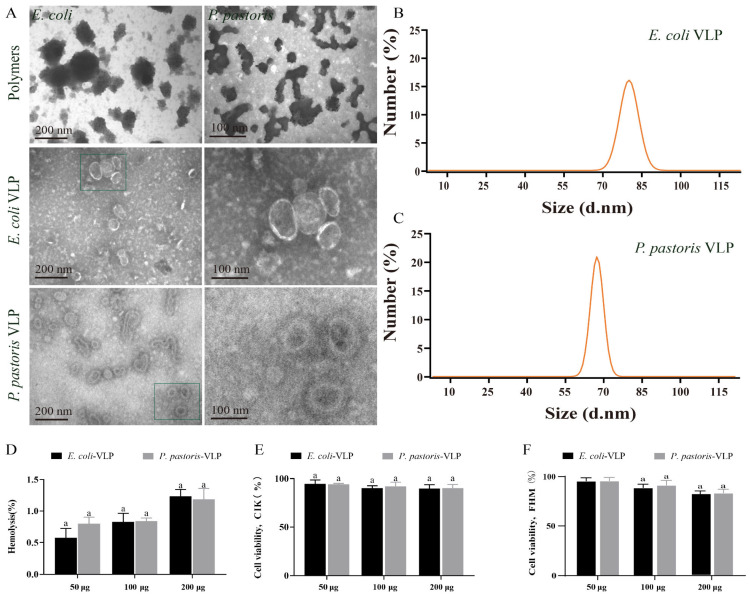
Detection of the appearance characteristics and cytotoxicity of *E. coli* VLPs and *P. pastoris* VLPs. (**A**) TEM image of polymers, *E. coli* VLPs, and *P. pastoris* VLPs. (**B**) *E. coli* VLP analysis using DLS. (**C**) *P. pastoris* VLP analysis using DLS. (**D**) Hemolysis study of *E. coli* VLPs and *P. pastoris* VLPs. Hemolytic activity of each group was detected with 2% grass carp red blood cells for 1 h at 37 °C. Data are expressed as mean ± SD (*n* = 3). (**E**) Toxicity assessment of *E. coli* VLPs and *P. pastoris* VLPs on CIK and (**F**) FHM cells by MTT assay. Data are expressed as mean ± SD (*n* = 3). Different lowercase letters in each group denote significant variations as calculated by the Kruskal–Wallis test followed by Dunn’s multiple comparisons test (*p* < 0.05).

TEM was utilized for morphological observation of the VLPs assembled under both condition A and condition B. TEM images displayed the polymers of *E. coli* and *P. pastoris* VLPs assembled under condition A, whereas the VLPs assembled in condition B exhibited more complete structures (Figure 2A). Furthermore, the TEM images confirmed that both *E. coli* VLPs and *P. pastoris* VLPs were homogeneous spherical VLPs (Figure 2A). These well-formed VLPs demonstrate potential as vaccine candidates. The DLS measurements revealed that the diameter and PDI of *E. coli* VLPs and *P. pastoris* VLPs were about 92 ± 10 nm and 75 ± 10 nm and, 0.28 ± 0.01 and 0.27 ± 0.01, respectively (Figure 2B,C). Hemolysis analysis and MTT analysis demonstrated that *E. coli* VLPs and *P. pastoris* VLPs did not exhibit significant toxicity towards grass carp red blood cells (Figure 2D), CIK cells (Figure 2E), or FHM cells (Figure 2F). These findings indicated the absence of substantial cellular toxicity, confirming the safety and tolerability of VLPs as vaccine antigens in grass carp. The assay results provided evidence of the morphological stability, uniform size, and low toxicity of these VLPs.

### 3.3. VLPs Enhance IgM Antibody Levels and Protective Efficacy While Alleviating Tissue Viral Load

**Figure 3:**
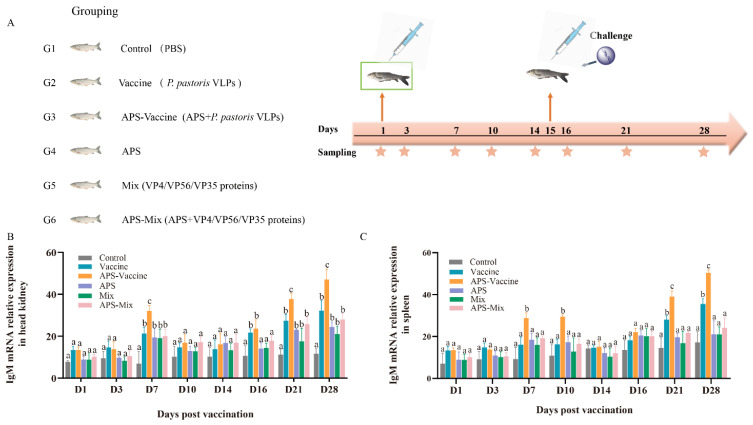
mRNA expression of IgM in grass carp head kidney and spleen. (**A**) Grass carp were divided into six groups (*n* = 50) and Vaccine, APS–Vaccine, APS, Mix, APS-Mix, and Control. (**B**) mRNA expression of IgM in the head kidney. (**C**) mRNA expression of IgM in the spleen.18S rRNA was used as the reference control gene. The different lowercase letters in each group (a, b, and c) denote significant variations calculated by the Kruskal–Wallis test followed by Dunn’s multiple comparisons test (*p* < 0.05). Sampling time points are represented by star.

The experimental flow and sampling time points are presented in the schedule (Figure 3A). The experimental results were compared among the following groups: Vaccine group, APS–Vaccine group, APS group, Mix group, APS–Mix group, and Control group (Figure 3A). The expression levels of IgM mRNA in the head kidney and spleen were measured by qRT–PCR after vaccination and viral challenge (Figure 3B,C). The results showed that the APS–Vaccine group exhibited the highest expression levels of IgM mRNA compared with the other groups, with a significant difference. Additionally, the APS–Vaccine group showed elevated levels of IgM mRNA at D28.

**Figure 4:**
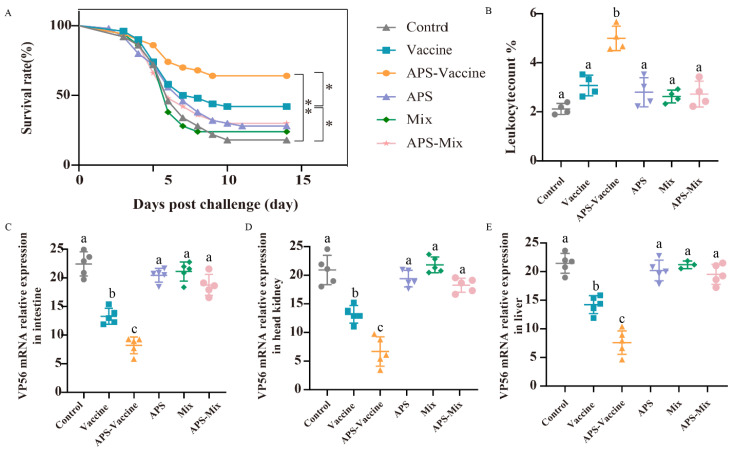
The survival rates, leukocyte numbers in blood and tissue viral loads after GCRV infection. (**A**) The survival rates of grass carp (*n* = 50) were monitored and calculated for 14 days after the GCRV 097 challenge. *p* values were calculated by Log-rank (Mantel-Cox) Test (* *p* < 0.05, ** *p* < 0.01). (**B**) The number of leucocytes per 1000 blood cells in the peripheral blood of grass carp. (**C**–**E**) Tissue viral loads of intestine (**C**), head kidney (**D**), and liver (**E**) on D28. The target gene for detecting GCRV was VP56, with β-actin and 18S rRNA used as reference control genes. Data are presented as mean ± SD (*n* = 4). The Kruskal–Wallis statistical test was used to identify significant variations in each group, which were further confirmed by Dunn’s multiple comparison test. Significant differences are denoted by lowercase letters (a, b, and c) in each group (*p* < 0.05).

Following the vaccination and challenge tests, the survival rates were as follows: 42% (Vaccine group), 62% (APS–Vaccine group), 28% (APS group), 24% (Mix group), 30% (APS–Mix group) and 22% (Control group). The APS–Vaccine group displayed a significantly higher survival rate than the control group (Figure 4A). Leukocyte number analysis revealed that the proportion of leukocytes in hematocrit was 5.1% in the APS–Vaccine group, which was significantly higher than in the control group, (2.24%) (Figure 4B). Furthermore, the APS–Vaccine group exhibited a significant reduction in GCRV viral content compared with the control group in various tissues (intestine, head kidney, and liver) (Figure 4C–E). Based on the antibody levels, survival rates, and tissue viral load test, it can be concluded that the APS–Vaccine group effectively increased IgM levels, improved survival rates, alleviated tissue virus load, and prevented viral infection.

### 3.4. VLPs Effectively Improve Serum Immune and Antioxidant Enzyme Activity against GCRV-II Infection

**Figure 5:**
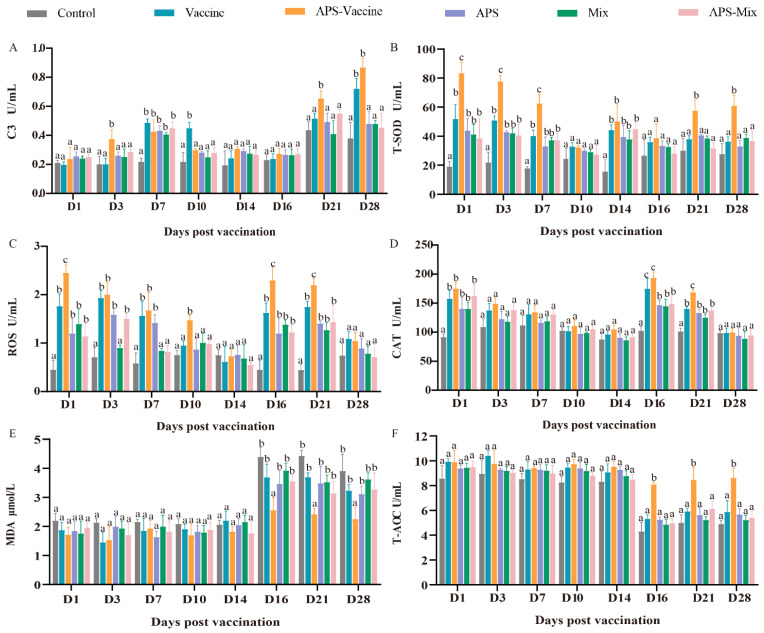
Serum biochemical parameters for innate immunity and antioxidant assays. (**A**) The assay for complement 3 content (C3). (**B**) The assay for total superoxide dismutase (T-SOD) activity. (**C**) The assay for ROS. (**D**) The assay for CAT. (**E**) The assay for MDA. (**F**) The assay for T-AOC. Serum biochemical indexes were determined using the corresponding specific commercial kits (Nanjing Jiancheng Bioengineering Institute, Nanjing, China). Data were presented as means ± SD (*n* = 4). Different lowercase letters in each group (a, b, and c) denote significant variations calculated by the Kruskal-Wallis statistics followed by Dunn’s multiple comparisons test (*p* < 0.05).

During the experiment, the six groups were evaluated to assess their ability to combat GCRV-II infection in grass carp, with eight-timepoints selected for sampling. The levels of C3, T-SOD, MDA, CAT, T-AOC, and ROS were measured as indicators. The content of C3 in the APS–Vaccine group showed a significant increase at D3, D7, D21, and D28, and was notably higher than the Control group at the same time points (Figure 5A). Meanwhile, after vaccination and viral injection, the T-SOD content gradually decreased due to the neutralization of virus clearance, and there was a significant difference between the APS–Vaccine group and the Control group at all sampling time points (Figure 5B). The ROS activity in the APS–Vaccine group was significantly higher than in the Control group on D1, D3, D7, D10, D16 and D21 (Figure 5C). The activity of CAT significantly increased on D1 D16, and D21 in the APS–Vaccine group (Figure 5D). The MDA activity in the APS–Vaccine group exhibited significant differences compared with the Control group on D16, D21, and D28 (Figure 5E). Likewise, T-AOC activity initially decreased after the viral challenge and then increased. In the APS–Vaccine group, the T-AOC activity significantly differed from that of the Control group on D16, D21, and D28 (Figure 5F). These findings suggest that the fish in the APS–Vaccine group had successfully activated various immune and antioxidant enzymes in their sera, which may have contributed to their ability to resist GCRV infection.

### 3.5. VLPs Facilitate the Expression of the Representative Immune Genes in Head Kidney

**Figure 6:**
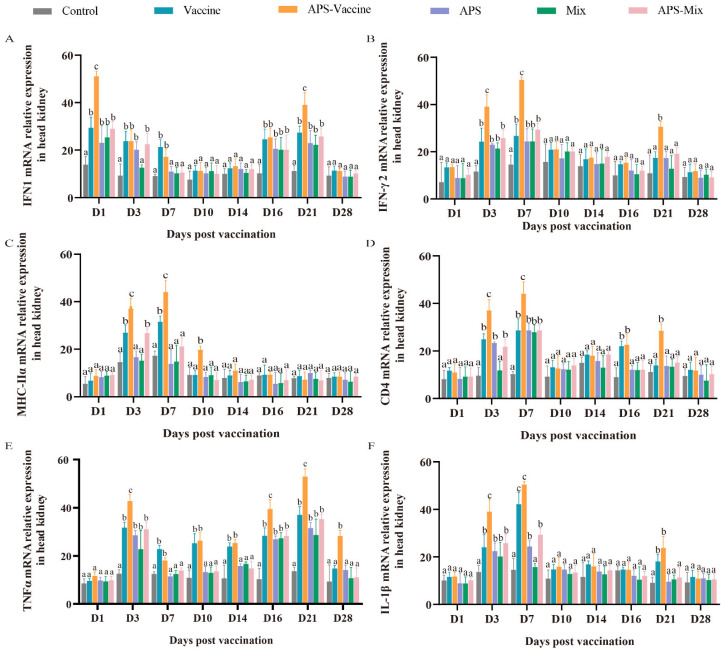
mRNA expression patterns of representative important immune regulation and effector genes in grass carp head kidney. (**A**–**F**) mRNA expressions of IFN1 (**A**), IFN-γ2 (**B**), MHC-IIα (**C**), CD4 (**D**), TNFα (**E**), and IL-1β (**F**) in the head kidney were determined by qRT-PCR. 18S rRNA and β-actin genes were used as reference control genes (*n* = 4). Different lowercase letters in each group (a, b, and c) denote significant variations calculated by the Kruskal–Wallis test followed by Dunn’s multiple comparisons test (*p* < 0.05).

To assess the effectiveness of VLP immunization, the mRNA expression levels of key immune genes (IFN1, IFN-γ2, MHC-IIα, CD4, TNFα, and IL-1β) were measured using qRT-PCR. Samples were collected from the head kidney at various timepoints after vaccination and challenge. The mRNA expression of IFN1 in the APS–Vaccine group showed a rapid upregulation on D1 and D21 and was significantly higher than that in the Control group on D1, D3, D7, D16, and D21 (Figure 6A). The mRNA expressions of IFN-γ2, MHC-IIα, CD4, and IL-1β in the APS–Vaccine group were significantly upregulated on D3 and D7 (Figure 6B–D,F). Additionally, the mRNA expression of TNFα in the APS–Vaccine group was higher than that in the other groups on D3, D16, D21, and D28 (Figure 6E). Overall, these results suggest that fish in the APS–Vaccine group had significantly induced the expression of key immune genes to combat GCRV infection.

### 3.6. VLPs Strengthen the Protective Effect and Reduce Tissue Lesions in Grass Carp Tissues

**Figure 7:**
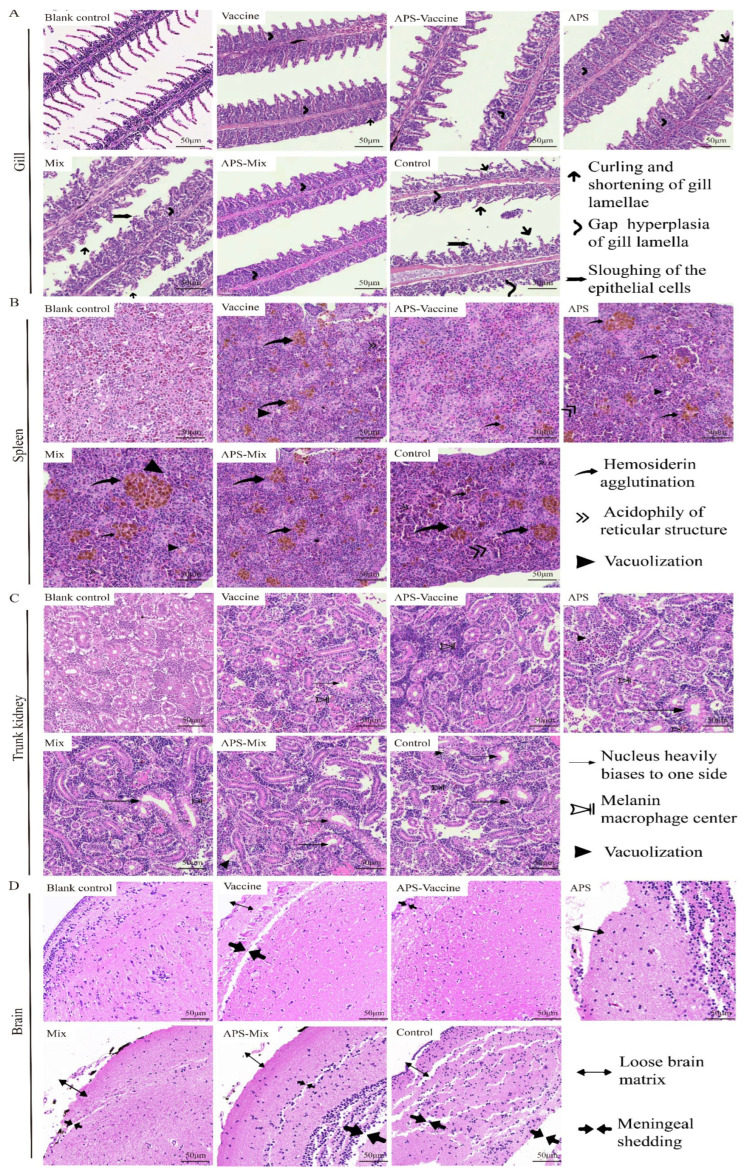
Histopathological examination of gill, spleen, trunk kidney, and brain tissues from grass carp post-vaccination and challenge. On D18, the gills, spleens, trunk kidneys, and brains were removed from grass carp to make sections and then stained with HE. Healthy grass carp tissues were used as the blank control. (**A**) Tissue scanning photographs of the gill. Gap hyperplasia of the gill lamella, curling, and shortening of gill lamellae, and sloughing of the epithelial cells were the three main lesion signs in gills. (**B**) Tissue scanning photographs of the spleen. Hemosiderin agglutination, acidophily of reticular structure, and vacuolization were the three main lesion signs in spleens. (**C**) Tissue scanning photographs of the trunk kidney. The nucleus heavily biases to one side of the cell, melanoma–macrophage centers, and vacuolization were the three main lesion signs in trunk kidneys. (**D**) Tissue scanning photographs of the brain. Loose brain matrix and meningeal shedding were the two main lesion signs in the brains.

On D18, gill, spleen, kidney, and brain tissues were collected to evaluate the degree of tissue damage. In the gill sections, the APS, Mix, and Control groups showed obvious symptoms such as curling and shortening of gill lamella, hyperplasia and gaps in gill lamella, and the sloughing of epithelial cells, compared with healthy grass carp gill tissues. However, the APS–Vaccine group exhibited milder symptoms (Figure 7A). In spleen sections, the diseased grass carp presented symptoms such as hemosiderin agglutination, acidophily of the reticular structure, and vacuolization. The other groups were more symptomatic than to the APS–Vaccine group (Figure 7B). In trunk kidney sections, healthy grass carp kidney tissues exhibited a compact structure and regular nucleus arrangement. The APS–Vaccine group showed the closest resemblance to a healthy status. The Control group exhibited the most severe lesions, characterized by nucleus displacement, melanin macrophage centers, and vacuolization (Figure 7C). In the brain tissue sections, the Control group showed a loose brain matrix and meninges shedding, whereas the APS–Vaccine group displayed a relatively tight and orderly structure similar to that of healthy grass carp (Figure 7D). Among the six experimental groups, the APS–Vaccine group exhibited a relatively milder degree of tissue lesions, indicating the superior protective effect of this regimen.

## 4. Discussion

GCHD remains a significant menace to the aquaculture sector, particularly affects grass carp, and has considerable economic ramifications. Previous research [28,29] has established that injection-based vaccination serves as an efficacious strategy to elicit protective immunity, owing to its immediate impact and successful immunization outcomes. Furthermore, it offers an effective and swift mechanism to develop an immunoprotective barrier before exposure to pathogens, thereby reducing disease mortality and enhancing survival rates [30]. The injection-based immunization approach enables a comprehensive examination and evaluation of the fish immune response. It has also been found that the coordinated delivery of vaccines and immune adjuvants can effectively enhance antigen-specific responses in vivo [31]. This study constructed a recombinant VLP vaccine augmented by adjuvants for injection-based immunization and used grass carp as the model to assess its effectiveness for inducing systemic immune responses. In future endeavors, the exploration of the impacts of oral and immersion administration of VLP vaccines is intended.

Over the past five decades, VLPs have precipitated significant advancements in vaccine development, with numerous vaccines, including Recombivax HB and Engerix-B for HBV, Gardasil, Cervarix, and Gardasil-9 for HPV, and Hecolin against HEV, already sanctioned for human use [32]. Studies have demonstrated that VLP vaccines, constructed via insect system expression, provided robust protection against GCRV infection [1]. Nonetheless, concerns have been raised about VLPs expressed and assembled in insect cells, as they may harbor residual host cell proteins and nucleic acids and thus pose potential safety risks [8,33].The technique for in vitro assembly is relatively straightforward, and the high immunogenicity and safety of successfully assembled VLPs have been validated [34,35]. The proteins VP4, VP56, and VP35 were successfully expressed and purified by *E. coli* and *P. pastoris* (Figure 1A,C). It has been hypothesized that the choice between the formation of spherical or tubular particles by VP4/VP56/VP35 proteins may be influenced by variations in ionic strength and pH. Therefore, we screened buffer conditions with different pH levels and salt concentrations to obtain VLPs that closely resembled natural virus structures (Figure S1E). It is anticipated that future enhancements in VLP assembly may be achieved using similar methods, rendering VLPs more suitable for vaccine or therapeutic use [36,37].

Owing to their structural congruence with natural viral entities, VLPs can inherently stimulate the immune system, eliminating the necessity for adjuvants. However, the incorporation of adjuvants in VLP vaccines can amplifying their immunogenicity and trigger specific forms of immune responses [7].As a vaccine adjuvant, APS can effectively bolster the natural immune defense mechanisms of animals, amplify the protective efficacy of vaccines, and enhance the growth performance of animals [38,39]. Despite the significant variation in the induction and modulation of immune genes related to the head kidney when utilizing APS alone, the results concerning survival rate, tissue load, and antioxidant capacity did not align with the projected outcomes. One rationalization for these results could be that the APS quantity deployed in this experiment might not have been the optimal concentration for inducing immune protection, thereby causing no phase elevation in the APS group. The immune effects in the Mix group also failed to provide more efficient protection, possibly due to the incomplete formation of the mixed proteins and lack of coating material, resulting in digestion of the exposed protein in vivo. Concurrently, the issues might also stem from the breeding environments and varietals [40].

There are three primary immunoglobulins in a fish’s immune system: IgM, IgD, and IgT [41]. Upon viral challenges, IgM antibodies play a critical role in activating the immune system by attaching to and neutralizing the virus [42]. In this study, the complex antigenic epitopes of VLPs stimulated the generation of a considerable amount of IgM antibodies in fish. The IgM antibody levels in the head kidney and spleen of the APS–Vaccine group were rapidly up-regulated during the pre-immune phase and subsequently exhibited a gradual down-regulation. These observations align with previous studies indicating that IgM antibody levels escalate swiftly during the initial phase of immune defense and decrease gradually as the virus is eradicated and the immune system is regulated [43].

The survival rate provides an intuitive reflection of a vaccine’s protective effect on the host, and it is a principal measure when assessing the vaccine’s immune efficacy [4]. Consistent with prior research [1], VLPs display substantial immunogenicity and robust protection against GCRV-II infection, leading to a 55% increase in the protection rate at two weeks post-GCRV-II challenge compared with the control group. In our investigation, both the Vaccine group and the APS–Vaccine group demonstrated significant differences compared with the Control group in terms of protection against the viral attack. Fish that received APS as a vaccine adjuvant exhibited superior viral resistance and significantly increased survival rates compared with fish in the other groups. Notably, a marked difference was observed between the Vaccine group and the APS–Vaccine group, thereby demonstrating the synergistic effect of APS and VLP co-immunization.

The viral load present in the primary tissues of grass carp can serve as an indicator of the severity of GCRV-II disease, making the analysis of viral load a potentially valuable predictor. Our results revealed substantial differences in tissue viral load within the intestines, head kidneys, and livers of the APS–Vaccine group compared with the other groups. Despite the significantly higher number of white blood cells observed in the APS–Vaccine group compared with the Control group, it is important to acknowledge that an elevation in white blood cells could also be a response to inflammation. Consequently, the count of white leukocytes cannot be solely used to accurately reflect the immune responses of grass carp within each experimental group.

In this investigation, VLPs were utilized as a vaccine, and various indices such as immunity and antioxidant serum enzyme activity were assessed. According to prior research, immune indicators such as C3 enzyme activity escalate rapidly to combat the virus following grass carp immunization [44,45]. Correspondingly, in this study, C3 significantly increased after the viral challenge to stimulate nonspecific immune production, clear viral residues, and repair bodily damage, which met our expectations. Similarly, antioxidative enzyme activity indicators, including MDA, ROS, T-AOC, and CAT exhibited significant differences in the APS–Vaccine group compared to the Control group, which is congruent with previous research findings [44].

MHC II molecules play a vital role during the onset of immune responses as they are responsible for presenting processed antigenic fragments to CD4+ T cells, and significantly influence humoral immunity [46,47]. A previous study reported an upregulation in MHC II mRNA expression levels in head kidneys following vaccination [20].

The rise in the count of MHC II^+^ cells at the infection site suggests that these cells enhance antibody responses by augmenting antigen presentation capacity and bolstering the associated immune response [48]. Our research findings revealed a significant upregulation in head and kidney CD4 and MHC-IIα in the APS–Vaccine group, indicating that the protective effect of VLPs might be due to their capability to stimulate both humoral and cellular immune responses. Frequently co-induced cytokines such as IL-1β and TNFα play a central role in modulating immune responses and inflammation which in turn can affect innate immune responses and lead to either defensive or pathological outcomes [49]. The rapid upregulation of IL-1β and TNFα mRNA expression in the APS–Vaccine group suggests that the treatment could more effectively mitigate the inflammatory response. IFN1 plays a crucial role in the host’s defense against viruses, and a significant increase in its mRNA expression levels was observed in the kidney after vaccination [50,51]. In our research, we noticed an upregulated trend at various time points for the mRNA expression of IFN1 and IFN-γ2 in the APS–Vaccine group, which illustrates the effective improvement in both cellular and humoral immunity to defend against GCRV infection in grass carp.

The potent immunogenicity of VP4/VP56/VP35 proteins in GCRV-II contributes to enhancing immune efficacy. We successfully expressed and purified these three proteins using both *E. coli* and *P. pastoris* expression systems. By mimicking the intracellular environment and adjusting the pH and salt concentration of the assembly buffer, we constructed *E. coli* VLPs and *P. pastoris* VLPs through efficient post-purification assembly. We investigated the structural features of VLPs, assessed their cytotoxicity on red blood cells, and examined their protective effects on grass carp through immunological assays, such as IgM antibody measurement, serum biochemical indicators, head kidney immune-related genes, and histopathological sections. Furthermore, we employed adjuvanted APS to establish an immune barrier and amplify the vaccine’s protective effects. Our findings underscored the vaccine’s effectiveness in safeguarding grass carp; thus, demonstrating its potential as a candidate vaccine and proposing a novel strategy for fish immunization.

## Figures and Tables

**Table 1:** Primer sequences and their designated application in this study.

Gene Name	Primer Sequence (5′−3′)	Amplicon Length (nt) and Primer Information
VP4	F: CGCGGATCCACTATCATGGGAAACGTC	1968
	R: CCG CTCGAGGTACACGACGTAAGACGG	Gene cloning
VP56	F: CGCGGATCC ATGGCCACTCGTGACAGCCGC	1539
	R: CCGCTCGAGTTACTTACAGC	Gene cloning
VP35	F: GCGTCGACATGGAATCAGCAAAACC	933
	R: CCGCTCGAGTTACTGTCCCTGG	Gene cloning
IL-1β	F: AAGTTCCCGCTTTGGAGAGTA	126
	R: GCCACATACCAGTCGTTCAGT	qRT-PCR
MHC-IIα	F: TACTACCAGATTCACTCGG	111
	R: CGGGTTCCAGTCAAAGAT	qRT-PCR
IFN1	F: GGTGAAGTTTCTTGCCCTGACCTTAG	173
	R: CCTTATGTGATGGCTGGTATCGGG	qRT-PCR
CD4	F: GTGCAGAGCTGCACTGCGACA	118
	R: GCACTATTTTGCCTCCTTCAGA	qRT-PCR
IgM	F: TGGAGCAACGGCACAGTATT	131
	R: TCTGGGGGTGCTAACAGGTA	qRT-PCR
β-actin	F: TCCTCACCGAGAGAGGCTAC	101
	R: CTGCTCGAAGTCAAGAGCCA	qRT-PCR
TNFα	F: CCAGCTCTTCCCAAACCAGT	126
	R: CCATCATCCTTCGCCCATGA	qRT-PCR
IFN-γ2	F: TCACAGTCAGGAAGCGAGTTC	100
	R: AAGGTTTGCGGCCCATCTTT	qRT-PCR
RIG-I	F: ACTACACTGAACACCTGCGGAA	108
	R: GCATCTTTAGTGCGGGCG	qRT-PCR
VP56	F: AGCAGGCTATTCATCACCAGT	107
	R: GTTCTAACGCTCACCGTCTTTTC	qRT-PCR
18S rRNA	F: ATTTCCGACACGGAGAGG	90
	R: CATGGGTTTAGGATACGCTC	qRT-PCR

Note: GenBank accession numbers are as follows: VP4, MN136091.1; VP56, MK675081.1; VP35, GU350748.1; β-actin, DQ835399.1; IgM, DQ417927.1; IFN1, AB196166.1; IFN-γ2, FJ766439.1; MHC-IIα, JF436931.1; CD4, KX033448.1; IL-1β, KX094935.1; TNFα, KX094934.1; 18S rRNA, EU047719.1.

## Data Availability

Not applicable.

## Supplementary Materials

The following supporting information can be downloaded at: https://www.mdpi.com/article/10.3390/vaccines11081373/s1, Figure S1. VP4, VP56, and VP35 gene induction, protein translation along with proper control (A–D) and screening of *E. coli* protein samples assembled under different pH conditions through GST pull down (E).
