# Supplementary material for: VP4/VP56/VP35 Virus-like Particles Effectively Protect Grass Carp (Ctenopharyngodon idella) against GCRV-II Infection
Source: Vaccines (Basel). 2023 Aug 16;11(8):1373. doi: 10.3390/vaccines11081373 (PMC10458301; doi:10.3390/vaccines11081373)
Supplement: Supplementary file 1 [file vaccines-11-01373-s001.zip › vaccines-2526447-supplementary.pdf]

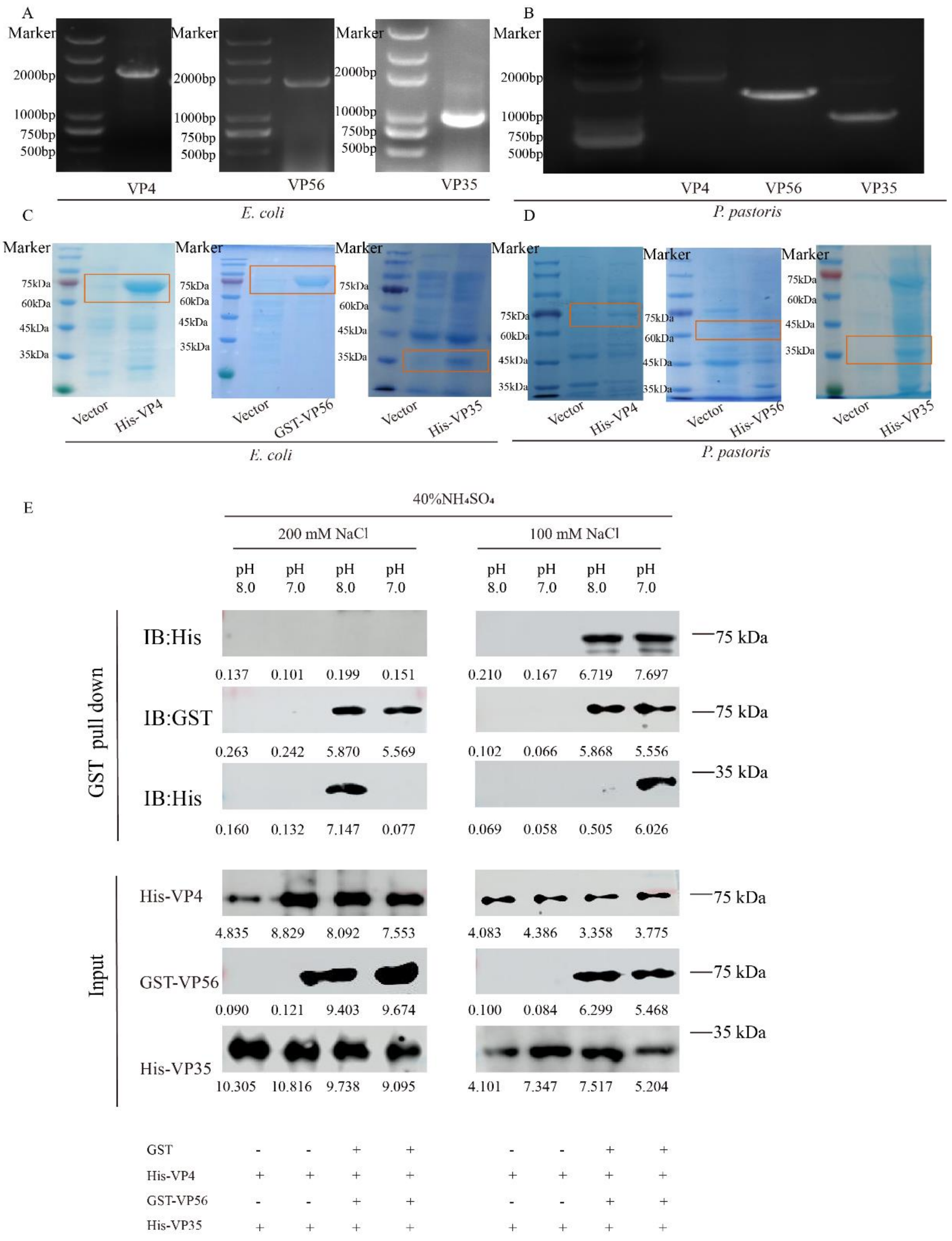

Figure S1. VP4, VP56, and VP35 gene induction, protein translation along with proper control and Screening of *E. coli* protein samples assembled under different pH conditions through GST pull down. (A-B) gene cloning of VP4/VP56/VP35 full-length. (C-D) SDS-PAGE analysis of the VP4, VP56, and VP35 proteins expression by *E. coli* and yeast, vector is control. (E) *E. coli* protein samples at pH 7.0 and 8.0, 100 mM and 200 mM NaCl 40% saturated $(NH_4)_2SO_4$ buffer was used for incubation at 4°C for 36 hours. Next, the samples were mixed with GST-bind resin for 2 hours at 4°C, followed by analysis with immunoblotting using anti-GST or anti-His antibodies.
